# Prognostic implications of echocardiographic and hemodynamic profiles in cirrhosis and high-output heart failure

**DOI:** 10.3389/fcvm.2026.1888790

**Published:** 2026-08-12

**Authors:** Satoshi Miyashita, Raquel Lamarche, Peter Oro, Roshan B. Patel, Karthik V, Rana Abualsaud, Yuichiro Yano, Toshio Naito, W. H. Wilson Tang, Daniel Burkhoff, Adriano R. Tonelli, Matthew T. Siuba

**Affiliations:** 1 Department of Critical Care Medicine, Cleveland Clinic, Integrated Hospital Care Institute, Cleveland, OH, United States; 2 Department of Cardiovascular Medicine, Heart, Vascular, and Thoracic Institute Cleveland Clinic Foundation, Cleveland, OH, United States; 3 Department of General Medicine, Juntendo University Faculty of Medicine, Tokyo, Japan; 4 Department of Internal Medicine, Cleveland Clinic Southpointe Hospital, Community Care Institute, Cleveland, OH, United States; 5 Department of Internal Medicine, Cleveland Clinic, Community Care Institute, Cleveland, OH, United States; 6 Mount Sinai Hospital, Institute of Critical Care Medicine, New York, NY, United States; 7 Division of Heart Failure, Hemoydnamics and MCS Research, Cardiovascular Research Foundation, New York, NY, United States; 8 Department of Pulmonary Medicine, Cleveland Clinic, Integrated Hospital Care Institute, Cleveland, OH, United States

**Keywords:** cardiac index, cirrhosis, echocardiogaphy, heart failure, hemodynamics, high-output cardiac failure, left atria enlargement, right heart catherterisation

## Abstract

**Introduction:**

Cirrhosis is a recognized cause of high-output heart failure (HOHF), yet the prognostic relevance of paired invasive hemodynamic and echocardiographic remodeling profiles in this population remains incompletely defined.

**Methods:**

We retrospectively analyzed 41 adults with cirrhosis-associated HOHF who underwent right heart catheterization between 2015 and 2023. HOHF was defined as thermodilution cardiac index (CI) ≥ 4.0 L/min/m^2^ with elevated filling pressures. The primary outcome was all-cause mortality.

**Results:**

During a median follow-up of 1.66 years, 18 patients died and 21 underwent liver transplantation. Mean CI was 5.5 ± 1.2 L/min/m^2^, mean MELD-Na score was 24 ± 9, and echocardiography demonstrated preserved left ventricular ejection fraction (65 ± 11%) with disproportionate left atrial enlargement relative to left ventricular volume, reflected by a mean LA/LV volume ratio of 0.84 ± 0.31. In univariable Cox regression analyses, higher LA/LV volume ratio, CI, and end-systolic elastance (Ees) were associated with increased mortality when modeled per 1-standard deviation increase, with hazard ratios of 2.13 (95% confidence interval, 1.20–3.79; *p* = 0.010), 1.64 (95% confidence interval, 1.12–2.42; *p* = 0.012), and 1.93 (95% confidence interval, 1.09–3.44; *p* = 0.024), respectively. In MELD-Na–adjusted Cox models, LA/LV volume ratio, CI, and Ees remained associated with mortality, with adjusted hazard ratios of 2.02 (95% confidence interval, 1.12–3.65; *p* = 0.019), 1.66 (95% confidence interval, 1.08–2.56; *p* = 0.021), and 1.92 (95% confidence interval, 1.09–3.39; *p* = 0.024), respectively. Similar directionality was observed in Firth penalized Cox models and Fine–Gray competing-risk models treating liver transplantation as a competing event.

**Discussion:**

In patients with cirrhosis-associated HOHF, disproportionate left atrial remodeling, elevated CI, and higher Ees were associated with mortality. These findings support further evaluation of integrated invasive hemodynamic and quantitative echocardiographic assessment for characterizing risk-related phenotypes in cirrhosis-associated HOHF.

## Introduction

High-output heart failure (HOHF) is an uncommon but distinct phenotype within the broader spectrum of heart failure (HF) (1). Because large HF registries typically do not differentiate HOHF from other subtypes, precise epidemiologic data are scarce; however, estimates suggest that HOHF may represent approximately 1%–2% of all HF cases, with higher prevalence in conditions such as advanced anemia, large arteriovenous fistulas, cirrhosis, obesity, or untreated hyperthyroidism (2–5).

HOHF is characterized by the clinical signs and symptoms of HF—including congestion and elevated filling pressures—despite abnormally high cardiac output at rest, frequently defined by a cardiac index (CI) exceeding 4.0 L/min/m^2^ or an absolute cardiac output above 8 L/min (6–9). Paradoxically, this elevated cardiac output is inadequate to meet peripheral metabolic demands. Alternatively, it may overwhelm the myocardium's capacity to handle the volume load, ultimately leading to overt HF (1, 2, 6).

Although the underlying pathophysiologic derangement—marked by reduced systemic vascular resistance (SVR), increased venous return, and increased stroke volume—culminates in signs and symptoms that resemble HF, HOHF remains understudied and is not well characterized in current guidelines (10, 11).

Cirrhosis is characterized by vasodilation, which leads to high output state (12). Cirrhosis-associated cardiopulmonary hemodynamics span a spectrum that includes hyperdynamic high-output physiology, volume-overload states, and pulmonary vascular disease such as portopulmonary hypertension. In this setting, RHC is particularly important because elevated pulmonary pressures may reflect high cardiac output or elevated filling pressures rather than pulmonary vascular remodeling alone. A recent consecutive liver transplant candidate screening cohort further highlighted the role of echocardiographic screening followed by RHC for characterizing portopulmonary hypertension in this population (13). However, whether cirrhosis-associated HOHF is accompanied by distinct patterns of cardiac chamber remodeling remains unclear. In particular, limited data exist regarding how paired invasive hemodynamic and quantitative echocardiographic profiles relate to clinical outcomes in patients with cirrhosis-associated HOHF. Building on prior work describing associations between invasive hemodynamic parameters and mortality in a broader HOHF cohort, the present study focused on echocardiographic chamber remodeling and its association with mortality in patients with cirrhosis-associated HOHF.

## Methods

This single-center retrospective study included adult (≥18 years) patients with cirrhosis who underwent elective right heart catheterization (RHC) at Cleveland Clinic Foundation between July 2015 and September 2023. Patients were eligible for inclusion if they had symptoms to suggest HF (i.e., leg edema or shortness of breath) and a thermodilution CI ≥ 4.0 L/min/m^2^ and elevated filling pressures—defined as right atrial pressure (RAP) ≥ 8 mmHg, pulmonary artery wedge pressure (PAWP) ≥ 15 mmHg, or mean pulmonary artery pressure (PAP) ≥ 20 mmHg—thus meeting the definition of HOHF (2, 3, 6, 8, 14). Patients on intravenous vasoactive medications or inotropic support were excluded. The present study was derived from the same institutional HOHF database used in our prior report and represents a cirrhosis subset of the previously published cohort, with overlap between the two study populations (8). Given the retrospective nature of the investigation, the Institutional Review Board approved the study with a waiver of informed consent (IRB #23-1022).

### Right heart catheterization

Hemodynamic measurements were obtained during clinically indicated RHC procedures following standard protocols as previously described (15). In brief, pressure transducers were zeroed at the midaxillary line in supine position. A 7.5-F pulmonary artery catheter was inserted through an 8.5-F introducer and advanced under fluoroscopic guidance into the pulmonary artery. The catheter position was confirmed by pressure waveform analysis and fluoroscopic imaging. Right-sided pressures — including RAP, systolic PAP, diastolic PAP, and mean PAP, as well as PAWP—were measured at end-expiration and averaged over at least three respiratory cycles using electronic calipers. RAP was recorded as the mean value across the respiratory cycles. PAWP was measured at the mid “a” wave, excluding the “v” wave (16). PAWP was confirmed by blood gas analysis (PAWP saturation > 94%), stable catheter on fluoroscopy, and characteristic waveform (17, 18). Cardiac output was determined using the thermodilution method repeated in triplicate to obtain values within 10%, and CI was calculated as cardiac output divided by body surface area (BSA) (19, 20).

### Echocardiographic measurements

Transthoracic echocardiograms performed within 90 days prior to RHC were reviewed. Echocardiographic acquisition and measurements were performed according to American Society of Echocardiography and European Association of Cardiovascular Imaging recommendations (21). Left-ventricular ejection fraction (LVEF) was calculated by the modified Simpson's method. The following parameters were abstracted: left ventricular (LV) diameter in diastole, LVEF, LV mass index, end-diastolic LV volume index, LV mass-to-volume ratio, right ventricular systolic function, right ventricular end-diastolic size, left atrial (LA) volume index, and the presence and severity of valvular heart disease. LA/LV volume ratio was defined as LA end-systolic volume divided by LV end-diastolic volume.

End-systolic LV elastance (Ees) was approximated as 0.9 × systolic blood pressure/end-systolic volume (22). Arterial elastance (Ea) was calculated as 0.9 × systolic blood pressure/stroke volume (22). Ventricular arterial coupling (VAC) was calculated as the ratio of Ea to Ees (22).

### Estimation of stressed blood volume

Stressed blood volume (SBV) was estimated with a validated simulation-based model of the cardiovascular system, as described previously (23). Briefly, the systemic and pulmonary circulations were modeled using series of resistors and capacitors, and cardiac chambers were represented by time-varying elastance elements. The model incorporated nine measured variables—heart rate, cardiac output, RAP, PAWP, systolic and PAPs, systolic and diastolic aortic pressures, and LVEF—to iteratively solve nonlinear differential equations via numerical integration. In cases with tricuspid regurgitation, backward flow was simulated using individualized effective regurgitant orifice area. A custom optimization algorithm was used to fit model output to patient-specific hemodynamic data, adjusting parameters such as right ventricle and LV elastance, diastolic stiffness, vascular compliance, and SBV. All SBV values were normalized to liters per 70 kg body weight.

### Outcomes

The primary outcome was all-cause mortality. Survival status and time to death or last follow-up were obtained from electronic medical records, with survival time defined as the interval from the date of RHC to the date of death or censoring.

### Statistical analysis

Continuous variables were expressed as mean ± standard deviation or median (interquartile range), according to their distribution. Categorical variables were summarized as frequencies and percentages. Between-group comparisons, when applicable, were performed using the Student t-test or Wilcoxon rank-sum test for continuous variables and the chi-square test or Fisher exact test for categorical variables.

Time-to-event analyses were performed from the date of RHC to death or administrative censoring. Cox proportional hazards regression was used to evaluate associations between hemodynamic and echocardiographic parameters and all-cause mortality. Continuous variables were modeled per 1-standard deviation increase to allow comparison across parameters measured on different scales. Univariable Cox models were first performed for exploratory screening, and multiple comparisons were adjusted using the false discovery rate.

For the primary adjusted analyses, LA/LV volume ratio, CI, and Ees were evaluated in parsimonious Cox models adjusted for MELD-Na. Each candidate marker was assessed in a separate model to limit model complexity. Firth penalized Cox regression was performed as a sensitivity analysis to account for the limited sample size and number of events. Because liver transplantation occurred frequently during follow-up, Fine–Gray competing-risk models were also performed, treating liver transplantation as a competing event for death.

The incremental discrimination of LA/LV volume ratio, CI, and Ees beyond MELD-Na was assessed using Harrell's C-statistic. For each candidate marker, analyses were performed using the corresponding marker-specific complete-case dataset, comparing MELD-Na alone with MELD-Na plus that marker. Changes in C-statistic were estimated using 2,000 paired bootstrap resamples. Nonlinear associations between LA/LV volume ratio, CI, Ees, and mortality were explored using MELD-Na–adjusted restricted cubic spline functions with 3 knots. Complete-case analyses were used for each model.

Two-sided *p*-values <0.05 were considered statistically significant. Statistical analyses were performed using R version 4.4.3 (R Foundation for Statistical Computing).

## Results

### Patient inclusion and characteristics

**Figure 1:**
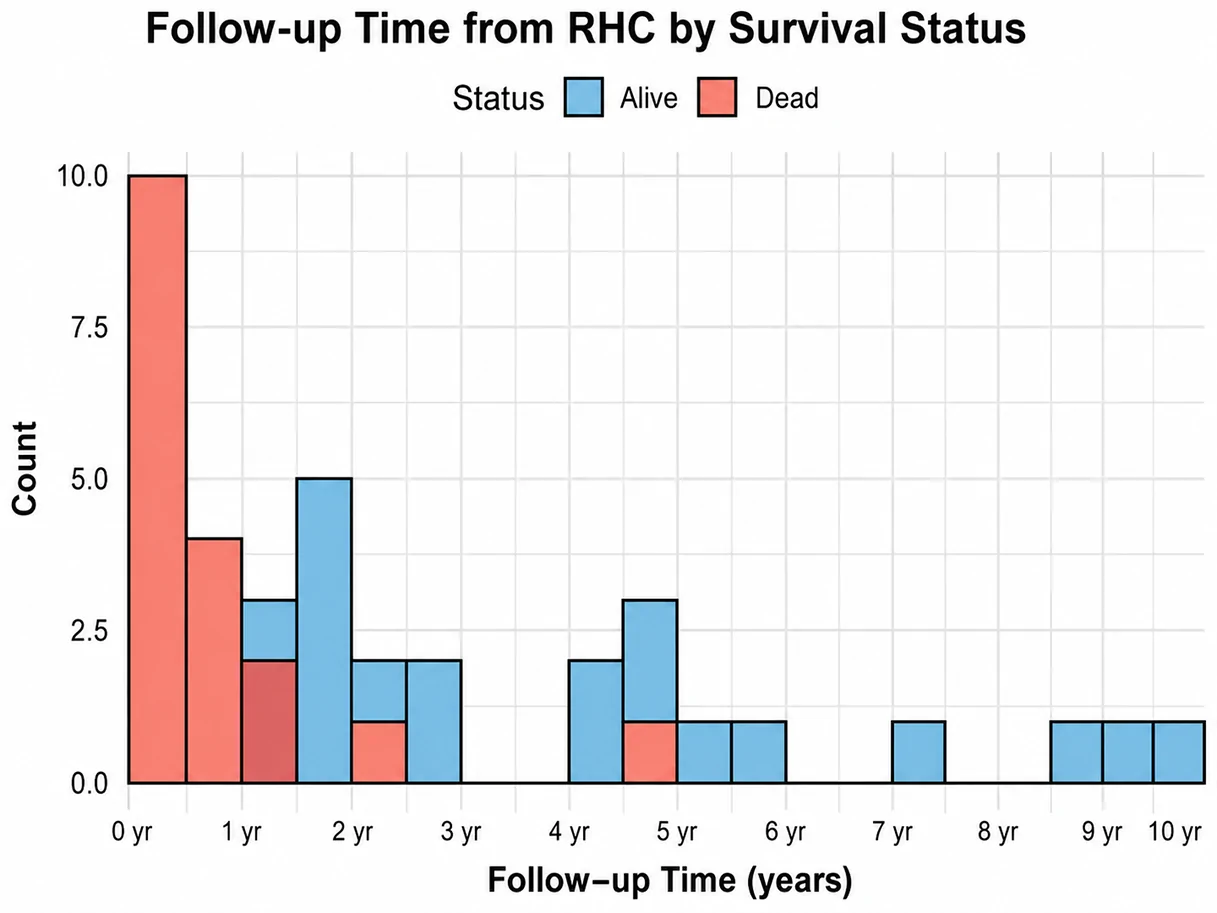
Distribution of follow-up time from right heart catheterization (RHC) stratified by survival status. Histogram showing the distribution of follow-up duration from the time of RHC to death or last follow-up, stratified by survival status. Follow-up time is displayed in 6-month intervals, with the *x*-axis labeled in years for clarity. Patients who died during follow-up (red) were predominantly clustered within the early follow-up period, whereas survivors (blue) demonstrated a broader distribution extending to longer follow-up durations. Overlapping regions indicate shared follow-up intervals between groups. RHC, right heart catheterization.

The final analytic cohort included 41 patients with cirrhosis-associated HOHF who had paired invasive hemodynamic and echocardiographic data (Supplementary Figure 1). During a median follow-up of 1.66 years (interquartile range, 0.55–4.47 years), 18 patients died and 21 underwent liver transplantation. The survival rates at 1 and 3 years were 65.9% and 57.2%, respectively. The distribution of follow-up time stratified by survival status is shown in Figure 1. The median interval between echocardiography and RHC was 5 days (interquartile range, 1–12 days), with 40 of 41 patients undergoing both assessments within 30 days.

Patient characteristics are shown in Table 1. The mean age was 55 ± 10 years, and 39% of patients were male. The majority were Caucasian (85.4%), with a body mass index (BMI) of 32.4 ± 8.1 kg/m^2^. Pre-existing diagnoses of HF were uncommon, with 7.3% having HF with a reduced LVEF and 9.8% HF with a preserved LVEF. Common comorbidities included hypertension (36.6%), diabetes (26.8%), and chronic kidney disease (22.0%). The mean estimated SBV was 2,844 ± 465 mL/70 kg, and mean CI was 5.5 ± 1.2 L/min/m^2^ (Table 2). Echocardiography showed LVEF of 65 ± 11%, LV mass index of 96 ± 34 g/m^2^, LV mass–volume ratio of 1.6 ± 0.4 g/mL, end-diastolic LV volume index of 60 ± 17 mL/m^2^, LA/LV volume ratio of 0.84 ± 0.31, and LA volume index of 50.4 ± 16.9 mL/m^2^ (Table 3).

**Table 1:** Basic characteristics.

Variable	Category	Overall	Alive	Dead	*P*-Value
n		41	23	18	
age		55.16 (9.94)	53.84 (10.62)	56.84 (9.01)	0.344
sex	Male	16 (39.0)	8 (34.8)	8 (44.4)	0.759
BMI		32.44 (8.07)	32.66 (8.11)	32.15 (8.24)	0.843
Race	Caucasian	35 (85.4)	19 (82.6)	16 (88.9)	0.442
	African	4 (9.8)	3 (13.0)	1 (5.6)	
	Hispanic	1 (2.4)	0 (0.0)	1 (5.6)	
	Other	1 (2.4)	1 (4.3)	0 (0.0)	
NYHA Class	I	4 (9.8)	2 (8.7)	2 (11.1)	0.565
	II	19 (46.3)	11 (47.8)	8 (44.4)	
	III	4 (9.8)	1 (4.3)	3 (16.7)	
	Missing	14 (34.1)	9 (39.1)	5 (27.8)	
Etiology of cirrhosis	NASH	11 (26.8)	7 (30.4)	4 (22.2)	0.25
	Alcohol	9 (22.0)	7 (30.4)	2 (11.1)	
	HCV	4 (9.8)	1 (4.3)	3 (16.7)	
	Other	17 (41.5)	8 (34.8)	9 (50.0)	
MELD-Na Score		24.23 (8.68)	25.36 (9.03)	22.76 (8.24)	0.361
Comorbidity	PH	10 (24.4)	5 (21.7)	5 (27.8)	0.936
	COPD	5 (12.2)	4 (17.4)	1 (5.6)	0.504
	Diastolic HF	4 (9.8)	3 (13.0)	1 (5.6)	0.825
	DM	11 (26.8)	8 (34.8)	3 (16.7)	0.905
	CKD	9 (22.0)	4 (17.4)	5 (27.8)	0.786
	HD	3 (7.3)	2 (8.7)	1 (5.6)	0.345
	HTN	15 (36.6)	8 (34.8)	7 (38.9)	0.677
	HLD	10 (24.4)	5 (21.7)	5 (27.8)	1
	Atrial fibrillation	3 (7.3)	1 (4.3)	2 (11.1)	0.477
Smoking	Active	0 (0)	0 (0)	0 (0)	0.446
	Never	23 (56.1)	10 (43.5)	13 (72.2)	0.354
	Former	18 (43.9)	13 (56.5)	5 (27.8)	0.128
	Midodrine	10 (24.4)	5 (21.7)	5 (27.8)	
	ACEi/ARB	1 (2.4)	1 (4.3)	0 (0.0)	
Medications	SGLT2i	0 (0)	0 (0)	0 (0)	0.936
	MRA	15 (36.6)	8 (34.8)	7 (38.9)	1
	BB	7 (17.1)	3 (13.0)	4 (22.2)	NA
	Diuretics	23 (56.1)	14 (60.9)	9 (50.0)	1

BMI, body mass index; NYHA, New York Heart Association; NASH, nonalcoholic steatohepatitis; HCV, hepatitis C virus; MELD-Na, model for end-stage liver disease sodium score; PH, pulmonary hypertension; COPD, chronic obstructive pulmonary disease; HF, heart failure; DM, diabetes mellitus; CKD, chronic kidney disease; HD, hemodialysis; HTN, hypertension; HLD, hyperlipidemia; ACEi, angiotensin-converting enzyme inhibitor; ARB, angiotensin II receptor blocker; SGLT2i, sodium-glucose cotransporter 2 inhibitor; MRA, mineralocorticoid receptor antagonist; BB, beta-blocker.

**Table 2:** Baseline invasive hemodynamic characteristics.

Variable	Overall	Alive	Dead	*P*-Value
HR (bpm)	83 (13)	81 (13)	86 (14)	0.279
MAP (mmHg)	86 (15)	85 (16)	87 (13)	0.682
RAP (mmHg)	15 (7)	16 (8)	15 (7)	0.83
sPAP (mmHg)	51 (17)	50 (15)	52 (19)	0.715
dPAP (mmHg)	28 (8)	28 (7)	29 (9)	0.846
mPAP (mmHg)	37 (11)	36 (9)	38 (13)	0.66
PAWP (mmHg)	21 (8)	21 (8)	21 (9)	0.793
SVR (dynes·s·cm⁻⁵)	537 (188)	542 (210)	529 (160)	0.827
PVR (L/min/m^2^)	1.5 (0.9)	1.4 (0.9)	1.5 (1.0)	0.767
CI (L/min/m^2^)	5.5 (1.2)	5.2 (0.8)	5.8 (1.4)	0.066
PAPi	1.8 (1.1)	1.8 (1.2)	1.8 (1.0)	0.949
Ees (mmHg/mL)	1.30 (0.82)	1.11 (0.46)	1.52 (1.09)	0.149
Ea (mmHg/mL)	0.64 (0.35)	0.60 (0.23)	0.70 (0.46)	0.393
VAC (Ea/Ees)	0.56 (0.28)	0.61 (0.33)	0.51 (0.20)	0.286
eSBV (mL/70 kg)	2,844 (465)	2,815 (502)	2,882 (423)	0.654

HR, heart rate; MAP, mean arterial pressure; RAP, right atrial pressure; sPAP, systolic pulmonary artery pressure; dPAP, diastolic pulmonary artery pressure; mPAP, mean pulmonary artery pressure; PAWP, pulmonary artery wedge pressure; SVR, systemic vascular resistance; PVR, pulmonary vascular resistance; CI, cardiac index; PAPi, pulmonary artery pulsatility index; Ees, end-systolic elastance; Ea, arterial elastance; VAC, ventricular-arterial coupling; eSBV, estimated stressed blood volume.

**Table 3:** Basic echocardiographic characteristics.

Variable	Category	Overall	Alive	Dead	*P*-Value
LVEF		64.95 (10.69)	65.17 (9.76)	64.67 (12.06)	0.882
LVIDd		4.94 (0.73)	4.89 (0.67)	5.01 (0.84)	0.647
LVM index		95.88 (34.18)	87.75 (31.01)	108.66 (36.12)	0.073
LV mass - volume ratio		1.61 (0.43)	1.49 (0.41)	1.82 (0.39)	0.029
LV volume index		60.49 (16.61)	60.58 (16.96)	60.37 (16.71)	0.969
RV systolic function	Normal	32 (78.0)	19 (82.6)	13 (72.2)	0.312
	Mildly	6 (14.6)	3 (13.0)	3 (16.7)	
	Moderate	2 (4.9)	0 (0.0)	2 (11.1)	
	Severe	1 (2.4)	1 (4.3)	0 (0.0)	
RV dilation	None	26 (63.4)	16 (69.6)	10 (55.6)	0.498
	Mild	5 (12.2)	3 (13.0)	2 (11.1)	
	missing	10 (24.4)	4 (17.4)	6 (33.3)	
LA volume index		50.43 (16.89)	46.49 (15.59)	56.51 (17.73)	0.128
LA/LV volume ratio		0.84 (0.31)	0.75 (0.24)	0.98 (0.36)	0.048
MR	None/Trace	3 (7.3)	2 (8.7)	1 (5.6)	0.574
	Mild	16 (39.0)	9 (39.1)	7 (38.9)	
	Moderate	18 (43.9)	11 (47.8)	7 (38.9)	
	Severe	2 (4.9)	1 (4.3)	1 (5.6)	
	missing	2 (4.9)	0 (0.0)	2 (11.1)	
TR	None/Trace	12 (29.3)	6 (26.1)	6 (33.3)	0.941
	Mild	20 (48.8)	12 (52.2)	8 (44.4)	
	Moderate	4 (9.8)	2 (8.7)	2 (11.1)	
	Severe	5 (12.2)	3 (13.0)	2 (11.1)	
AS	None	29 (70.7)	18 (78.3)	11 (61.1)	0.486
	Mild	7 (17.1)	3 (13.0)	4 (22.2)	
	missing	5 (12.2)	2 (8.7)	3 (16.7)	
AR	None/Trace	22 (53.7)	13 (56.5)	9 (50.0)	0.098
	Mild	11 (26.8)	8 (34.8)	3 (16.7)	
	Moderate	3 (7.3)	2 (8.7)	1 (5.6)	
	Severe	2 (4.9)	0 (0.0)	2 (11.1)	
	missing	3 (7.3)	0 (0.0)	3 (16.7)	
LV diastolic dysfunction	Normal	7 (17.1)	5 (21.7)	2 (11.1)	0.176
	Grade I	6 (14.6)	5 (21.7)	1 (5.6)	
	Grade II	9 (22.0)	6 (26.1)	3 (16.7)	
	other	18 (43.9)	7 (30.4)	11 (61.1)	
	NA	1 (2.4)	0 (0.0)	1 (5.6)	
lateral E/e′		12.96 (5.42)	11.94 (4.98)	14.90 (5.95)	0.166
septal E/e′		16.41 (6.24)	16.48 (7.25)	16.29 (4.18)	0.939
E/e′		14.74 (5.47)	14.27 (5.84)	15.60 (4.93)	0.55

LVEF, left ventricular ejection fraction; LVIDd, left ventricular internal diameter in diastole; LVM, left ventricular mass; LV, left ventricle; RV, right ventricle; LA, left atrium; MR, mitral regurgitation; TR, tricuspid regurgitation; AS, aortic stenosis; AR, aortic regurgitation; E/e′, ratio of early mitral inflow velocity to mitral annular early diastolic velocity; lateral E/e′, lateral annulus-derived E/e′; septal E/e′, septal annulus-derived E/e′.

### Survival analysis

**Figure 2:**
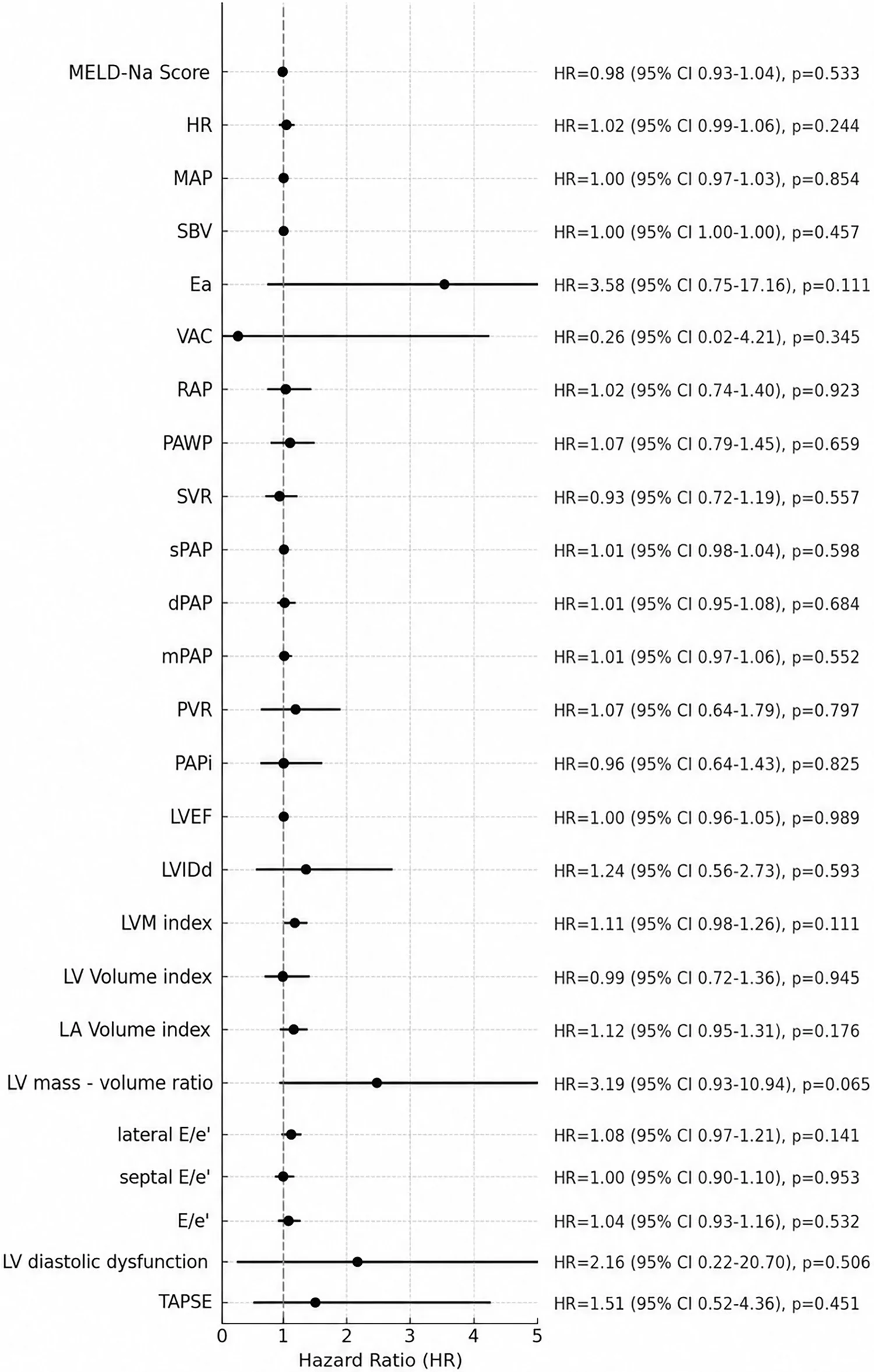
Exploratory univariable associations of noncandidate hemodynamic and echocardiographic parameters with mortality. Forest plot showing univariable Cox regression analyses for exploratory hemodynamic and echocardiographic parameters other than LA/LV volume ratio, CI, and Ees. Hazard ratios are shown per 1-SD increase for continuous variables to allow comparison across parameters measured on different scales. Points represent hazard ratios, and horizontal bars represent 95% confidence intervals. False-discovery-rate correction was applied across exploratory univariable comparisons. CI, confidence interval; dPAP, diastolic pulmonary artery pressure; Ea, arterial elastance; E/e′, ratio of early mitral inflow velocity to mitral annular early diastolic velocity; Ees, end-systolic elastance; HR, heart rate when listed as a variable and hazard ratio when reported as an effect estimate; LA, left atrial; LA volume index, left atrial volume index; LA/LV, left atrial-to-left ventricular; lateral E/e′, lateral annulus-derived E/e′; LV, left ventricular; LV diastolic dysfunction, left ventricular diastolic dysfunction; LVEF, left ventricular ejection fraction; LVIDd, left ventricular internal diameter in diastole; LVM index, left ventricular mass index; LV mass-volume ratio, left ventricular mass-to-volume ratio; LV volume index, left ventricular volume index; MAP, mean arterial pressure; MELD-Na, Model for End-Stage Liver Disease–Sodium; mPAP, mean pulmonary artery pressure; PAPi, pulmonary artery pulsatility index; PAWP, pulmonary artery wedge pressure; PVR, pulmonary vascular resistance; RAP, right atrial pressure; SBV, stressed blood volume; septal E/e′, septal annulus-derived E/e′; sPAP, systolic pulmonary artery pressure; SVR, systemic vascular resistance; TAPSE, tricuspid annular plane systolic excursion; VAC, ventricular-arterial coupling.

In univariable Cox regression analyses, higher LA/LV volume ratio, CI, and Ees were associated with increased mortality. The corresponding hazard ratios were 2.13 for LA/LV volume ratio (95% CI, 1.20–3.79; *p* = 0.010), 1.64 for CI (95% CI, 1.12–2.42; *p* = 0.012), and 1.93 for Ees (95% CI, 1.09–3.44; *p* = 0.024). These associations did not remain statistically significant after false-discovery-rate correction. Other hemodynamic and echocardiographic parameters, including filling pressures, stressed blood volume, systemic vascular resistance, pulmonary vascular resistance, LVEF, LV mass index, LA volume index, Ea, and VAC, were not significantly associated with mortality in exploratory univariable analyses (Figure 2).

**Figure 3:**
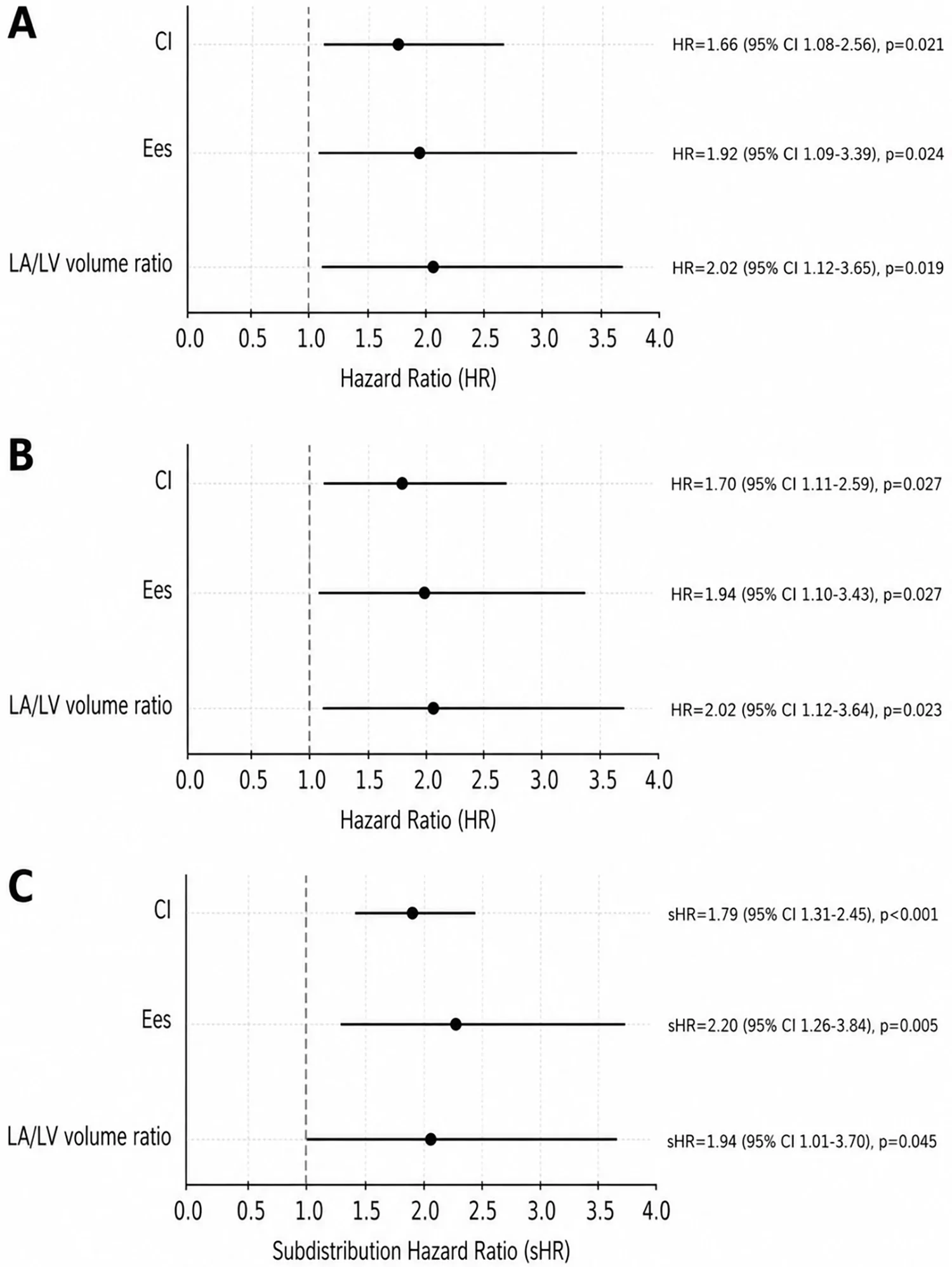
Exploratory adjusted associations of candidate hemodynamic and echocardiographic markers with mortality. Forest plots showing associations of CI, Ees, and LA/LV volume ratio with mortality. All estimates are shown per 1-SD increase in each variable. **(A)** Cox proportional hazards models adjusted for MELD-Na. **(B)** Firth penalized Cox models adjusted for MELD-Na. **(C)** Fine–gray competing-risk models adjusted for MELD-Na, treating liver transplantation as a competing event. Points represent hazard ratios or subdistribution hazard ratios, and horizontal bars represent 95% confidence intervals. CI, cardiac index; Ees, end-systolic elastance; HR, hazard ratio; LA, left atrial; LV, left ventricular; MELD-Na, Model for End-Stage Liver Disease–Sodium; sHR, subdistribution hazard ratio.

In MELD-Na–adjusted Cox models, higher LA/LV volume ratio, CI, and Ees were each associated with increased mortality. The magnitude and direction of these associations were similar in Firth penalized Cox models and in Fine–Gray competing-risk models treating liver transplantation as a competing event (Figure 3).

**Figure 4:**
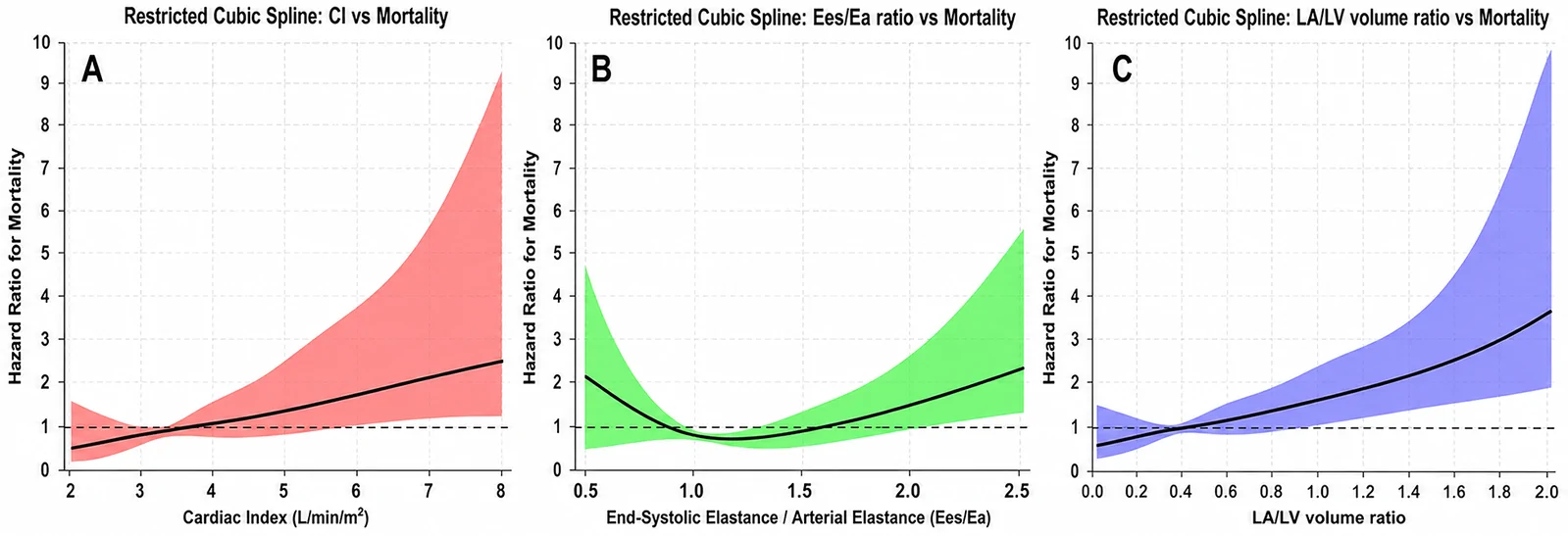
RCS analyses hemodynamic parameters for all-cause mortality. Panel **(A)** unadjusted RCS curve for cardiac index. Panel **(B)** unadjusted RCS curve for End-systolic elastance. Panel **(C)** unadjusted RCS curve for LA/LV left atrial-to-left ventricular volume ratio. RCS, restricted cubic spline curve. LA/LV, left atrial-to-left ventricular.

In exploratory discrimination analyses, addition of LA/LV volume ratio, CI, or Ees to MELD-Na numerically increased Harrell's C-statistic; however, the changes in C-statistic were not statistically significant by paired bootstrap analysis. Harrell's C-statistic increased from 0.49 to 0.69 with addition of LA/LV volume ratio (*Δ*C 0.19; bootstrap 95% CI, −0.05 to 0.34; *p* = 0.193), from 0.51 to 0.64 with addition of CI (*Δ*C 0.13; bootstrap 95% CI, −0.01 to 0.29; *p* = 0.102), and from 0.55 to 0.59 with addition of Ees (*Δ*C 0.04; bootstrap 95% CI, −0.06 to 0.22; *p* = 0.371). MELD-Na–adjusted restricted cubic spline analyses did not demonstrate strong evidence of nonlinearity for LA/LV volume ratio, cardiac index, or Ees (all *p* for nonlinearity > 0.05) (Figure 4).

## Discussion

To our knowledge, this is the first study to characterize paired echocardiographic remodeling and invasive hemodynamic profiles in patients with cirrhosis-associated HOHF and to examine their associations with mortality. In this retrospective cohort of severely ill patients with advanced liver disease, several key findings emerged. First, LA enlargement was disproportionately greater relative to LV volume, as reflected by an increased LA/LV volume ratio. Higher LA/LV volume ratio was associated with increased mortality in MELD-Na–adjusted Cox models, with similar findings in Firth penalized Cox and Fine–Gray competing-risk analyses. Second, higher CI and Ees were also associated with mortality in MELD-Na–adjusted models and showed similar directionality in sensitivity analyses. In contrast, other echocardiographic and hemodynamic indices—including LVEF, SBV, SVR, and filling pressures—were not associated with mortality.

Our findings underscore the high morbidity and mortality associated with HOHF and cirrhosis, consistent with prior studies. In one series, five-year mortality varied by etiology, ranging from 19% in obesity-related HOHF to nearly 60% in those with liver disease or shunt-associated HOHF (7). In our cohort, patients had a survival of 65.9% at one year and 57.2% at 3 years, reinforcing that patients with cirrhosis and HOHF carry a high risk of mortality.

In patients with cirrhosis, cardiac remodeling is thought to result from a combination of factors, including reduced SVR due to vasodilation, expanded intravascular volume, elevated metabolic demands, and the effects of circulating cytokines and vasoactive hormones (24). Echocardiographic studies of cirrhotic cardiomyopathy have described several structural and functional changes—such as increased LA size, LV end-diastolic dimensions, elevated pulmonary pressures, and indices of diastolic dysfunction—with weak associations to liver disease severity and prognosis (25, 26). However, these descriptions largely reflect cirrhosis as a whole, and echocardiographic or invasive hemodynamic features specific to patients with high-output physiology have not been well defined. In this context, our study characterizes these features in a cohort of patients with cirrhosis and HOHF and additionally evaluates their associations with mortality.

In the present study, patients with HOHF confirmed by invasive hemodynamic assessment exhibited a marked disproportion between LA and LV chamber enlargement, with LA volume increasing to a substantially greater extent than LV volume. This pattern differs from the chamber remodeling typically described in cirrhotic cardiomyopathy, in which LV dilatation and diastolic abnormalities are prominent. When examined as a continuous variable, the LA/LV volume ratio was associated with mortality and appeared to be more closely related to outcomes than other echocardiographic measures evaluated in this cohort. In support of these findings, prior studies have demonstrated that an elevated LA/LV volume ratio carries physiologic and prognostic relevance across several settings (27). First, the LA/LV volume ratio has been described in hyperdynamic circulatory states, such as high-intensity exercise, where it correlates with peak VO₂ and indices of filling pressure (28). Beyond hyperdynamic states, the prognostic relevance of the LA/LV volume ratio has also been demonstrated in HF and structural heart disease (29). In severe functional mitral regurgitation, it has been shown to stratify mortality risk, with one study reporting a hazard ratio of 1.37 (95% CI, 1.13–1.65) when dichotomized at 0.56 (29). Collectively, these observations indicate that the LA/LV volume ratio reflects the balance between atrial and ventricular remodeling under chronic volume loading conditions, providing integrated information about diastolic burden and chamber interaction that is not captured by standard echocardiographic indices.

Prior work has proposed that an increased LA/LV volume ratio reflects a limitation in LV dilatation due to chronic hypertrophy and fibrosis, leaving the LA as the principal reservoir to accommodate chronic volume and pressure loading (24, 25). Cirrhosis can contribute to this pattern through multiple pathways, including cytokine release, activation of vasoactive hormones, mitochondrial dysfunction, volume overload, systemic vasodilation, and increased peripheral oxygen demand (24, 26, 30). Within this pathophysiologic context, higher LA/LV volume ratio was associated with mortality in MELD-Na–adjusted models and showed consistent directionality across sensitivity analyses.

Using the HARVI platform, we were able to quantitatively estimate SBV, demonstrating that both SBV and cardiac filling pressures were elevated in patients with HOHF. In healthy individuals, estimated SBV is typically approximately 1,700–1,800 mL per 70 kg, whereas SBV in our cohort averaged 2,844 ± 465 mL per 70 kg, consistent with marked intravascular volume expansion. HARVI-derived SBV has been shown across multiple HF conditions—including HFpEF, HF with reduced ejection fraction, severe tricuspid regurgitation, pulmonary hypertension, and cardiogenic shock—to provide a quantitative index of congestion and prognostic information (23). However, in our cohort, neither SBV nor filling pressures were associated with mortality, representing a notable departure from prior work. This dissociation suggests that, in cirrhosis-associated HOHF, risk may not be fully captured by conventional measures of hemodynamic congestion alone.

Our findings also showed that elevated CI and Ees were associated with increased mortality in univariable analysis. Unlike LVEF—which is highly load dependent—CI and Ees reflect dynamic cardiac performance and may be more sensitive markers of physiologic stress in HOHF. Prior studies have reported varied relationships between CI and outcomes, including a U-shaped association in cirrhosis and adverse renal effects at high CI in advanced HF (9, 31–33). These associations persisted after adjustment for MELD-Na and showed similar directionality in Firth penalized Cox models and Fine–Gray competing-risk analyses. The larger subdistribution hazard estimate for CI in the Fine–Gray model may reflect the influence of liver transplantation as a competing event on the cumulative incidence of death, particularly in this small transplant-enriched cohort, and should therefore be interpreted cautiously. In MELD-Na–adjusted restricted cubic spline analyses, CI and Ees did not show strong evidence of nonlinearity, suggesting that their associations with mortality were not driven by a clear threshold effect. Together, elevated CI and Ees may reflect a hyperdynamic, high-stress cardiovascular phenotype in cirrhosis-associated HOHF rather than preserved cardiovascular reserve.

Despite their observed associations with mortality, LA/LV volume ratio, CI, and Ees are not routinely assessed during standard echocardiography. Our findings indicate that incorporating these parameters—particularly in patients with cirrhosis and suspected HOHF—may provide additional phenotypic information and warrants further evaluation for risk assessment (34). Evidence supporting therapies in cirrhosis with HOHF remains limited, although SGLT2 inhibitors have shown promise in observational studies, with one large propensity-matched analysis demonstrating reduced rates of serious liver-related complications (35). Notably, none of the patients in our cohort were receiving SGLT2 inhibitors. As cirrhotic cardiomyopathy remains underrecognized despite its high prevalence, these results underscore the need to enhance cardiovascular assessment and to explore risk-guided therapeutic strategies in patients with cirrhosis and HOHF.

## Limitations

Several limitations merit consideration. First, this was a retrospective, single-center study from a tertiary referral and transplant center, and the cohort consisted of patients with advanced liver disease; therefore, the findings may not be generalizable to broader cirrhosis populations or patients with less severe illness. Second, the sample size and number of events were limited, increasing the risk of model overfitting. We therefore used parsimonious models, Firth penalized Cox regression, and Fine–Gray competing-risk models as sensitivity analyses; however, all adjusted and discrimination analyses should be interpreted as exploratory. Third, liver transplantation occurred frequently during follow-up and represents an important competing event for death. Although competing-risk analyses were performed, residual bias related to transplant selection, timing, and candidacy cannot be excluded. Fourth, this cohort was derived from the same institutional HOHF database as our prior report and represents a cirrhosis-focused subset with overlapping patients; therefore, the present findings should be interpreted as extending prior invasive hemodynamic work by incorporating quantitative echocardiographic remodeling measures rather than as an entirely independent cohort. Fifth, echocardiography and RHC were not always performed on the same day, although the median interval was short. Sixth, medication exposure and treatment changes over time were not standardized, and residual confounding from diuretic use, beta-blockers, vasoactive therapies, liver disease severity, and transplant candidacy may remain. In addition, exploratory discrimination analyses were limited by the small sample size, marker-specific complete-case datasets, and low baseline discrimination of MELD-Na alone, which limits the interpretability of the observed changes in Harrell's C-statistic. Finally, the study focused on all-cause mortality and lacked external validation. Future multicenter studies are needed to validate these findings and evaluate additional outcomes such as HF hospitalization, liver-related complications, and post-transplant outcomes.

Nonetheless, this study provides insight into a population that remains poorly defined. By integrating invasive hemodynamics with quantitative echocardiographic assessment, we identified a pattern of disproportionate atrial remodeling and observed that LA/LV volume ratio was associated with mortality despite the lack of association between conventional congestion measures and mortality. These observations support further investigation of comprehensive cardiac evaluation in patients with cirrhosis and suspected HOHF and provide a rationale for future studies to validate these findings and clarify whether these measures can inform clinical risk assessment or therapeutic decision-making.

## Conclusions

In patients with cirrhosis-associated HOHF confirmed by invasive hemodynamic assessment, we identified a distinct pattern of disproportionate LA dilation relative to LV volume. Higher LA/LV volume ratio, CI, and Ees were associated with increased mortality, whereas conventional measures such as LVEF, filling pressures, SVR, and SBV were not. These findings suggest that integrating invasive hemodynamics with quantitative echocardiographic remodeling assessment may help identify higher-risk phenotypes in cirrhosis-associated HOHF and support further investigation in larger, externally validated cohorts.

## Data Availability

The raw data supporting the conclusions of this article will be made available by the authors, without undue reservation.
