# Supplementary material for: Prognostic implications of echocardiographic and hemodynamic profiles in cirrhosis and high-output heart failure
Source: Front Cardiovasc Med. 2026 Aug 12;13:1888790. doi: 10.3389/fcvm.2026.1888790 (PMC13506855; doi:10.3389/fcvm.2026.1888790)

Supplemental Figure 1. Study cohort derivation.

Flow diagram showing derivation of the final analytic cohort. From the institutional high-output heart failure cohort (n = 248), 69 patients with cirrhosis were identified. Among these, 25 patients were excluded because transthoracic echocardiography was not performed within 90 days of right heart catheterization. Of the remaining 44 patients who met echocardiographic timing criteria, 3 were excluded because of insufficient echocardiographic data. The final study cohort included 41 patients with cirrhosis-associated high-output heart failure and paired invasive hemodynamic and echocardiographic data. HOHF: high-output heart failure.

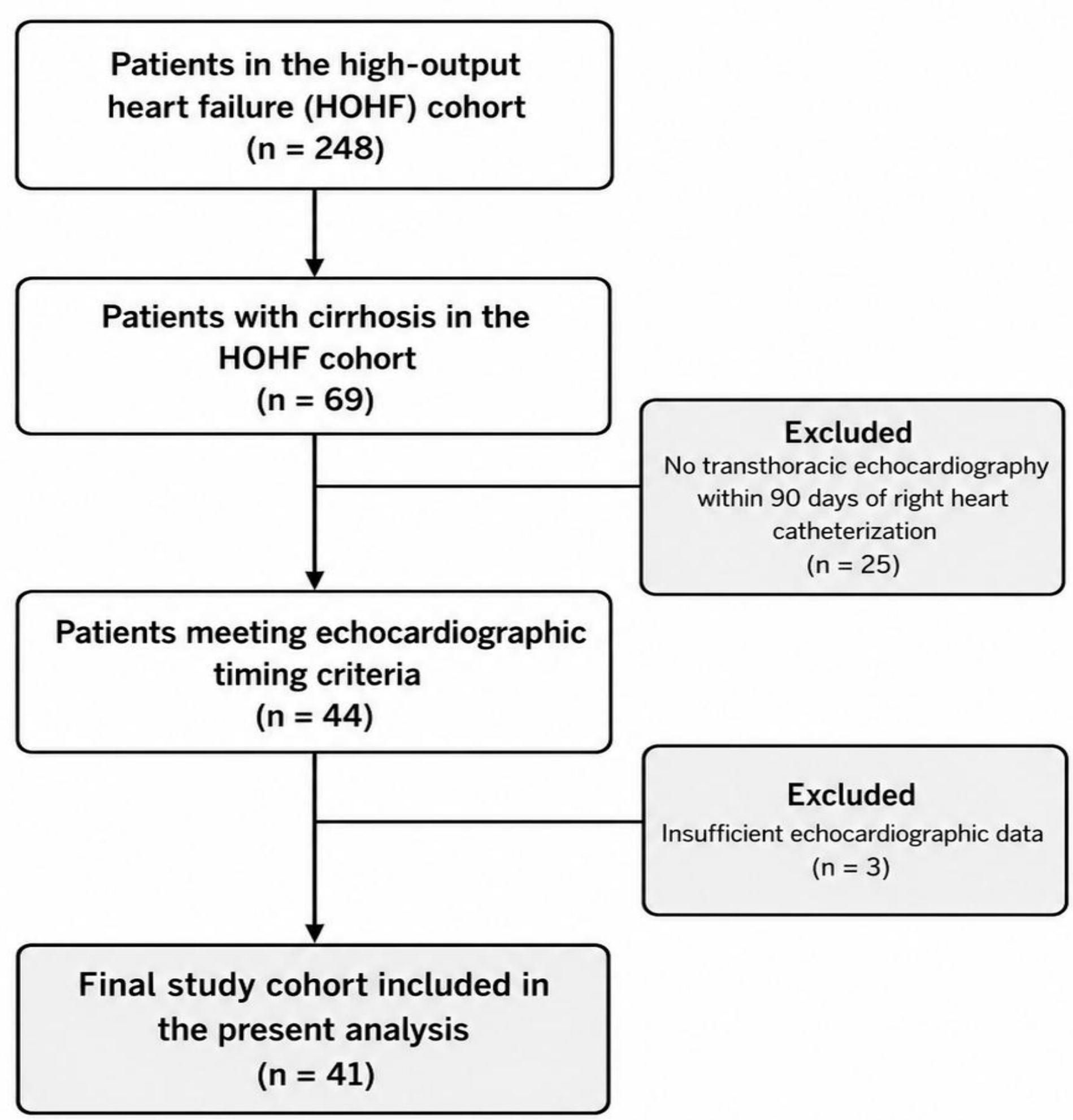

Supplement: Supplementary file 1 [file Image1.pdf]
